# An Automation System for Controlling Streetlights and Monitoring Objects Using Arduino

**DOI:** 10.3390/s18103178

**Published:** 2018-09-20

**Authors:** Zain Mumtaz, Saleem Ullah, Zeeshan Ilyas, Naila Aslam, Shahid Iqbal, Shuo Liu, Jehangir Arshad Meo, Hamza Ahmad Madni

**Affiliations:** 1 Department of Computer Science, Khwaja Fareed University of Engineering and Information Technology, Rahim Yar Khan 64200, Pakistan; zainmumtaz007@gmail.com (Z.M.); saleem.ullah@kfueit.edu.pk (S.U.); zeeshanilyas002@gmail.com (Z.I.); nailaaslam163@gmail.com (N.A.); 2 State Key Laboratory of Millimeter Waves, Department of Radio Engineering, Southeast University, Nanjing 210096, China; shahid@seu.edu.cn (S.I.); liushuo.china@seu.edu.cn (S.L.); 3 Department of Electrical Engineering, COMSATS University Islamabad, Islamabad 45550, Pakistan; jehangir@ciitsahiwal.edu.pk; 4 Department of Computer Engineering, Khwaja Fareed University of Engineering and Information Technology, Rahim Yar Khan 64200, Pakistan

**Keywords:** Arduino, automation, energy consumption, low-cost, microcontroller, open source, streetlights, smart homes, sensors

## Abstract

We present an Arduino-based automation system to control the streetlights based on solar rays and object’s detection. We aim to design various systems to achieve the desired operations, which no longer require time-consuming manual switching of the streetlights. The proposed work is accomplished by using an Arduino microcontroller, a light dependent resistor (LDR) and infrared-sensors while, two main contributions are presented in this work. Firstly, we show that the streetlights can be controlled based on the night and object’s detection. In which the streetlights automatically turn to DIM state at night-time and turn to HIGH state on object’s detection, while during day-time the streetlights will remain OFF. Secondly, the proposed automated system is further extended to skip the DIM condition at night time, and streetlights turn ON based on the objects’ detection only. In addition, an automatic door system is introduced to improve the safety measurements, and most importantly, a counter is set that will count the number of objects passed through the road. The proposed systems are designed at lab-scale prototype to experimentally validate the efficiency, reliability, and low-cost of the systems. We remark that the proposed systems can be easily tested and implemented under real conditions at large-scale in the near future, that will be useful in the future applications for automation systems and smart homes.

## 1. Introduction

Automation systems [1,2] have the advantage over the manual systems because it increases the productivity, efficiency and reliability, and minimizes the usage of resources to save energy, and reduce the operating cost etc. These automation systems play an essential role in the term “smart home” [3,4,5,6,7,8,9] to make our daily life more comfortable, and to facilitate users from ceiling fans to ovens, and in other applications. Among all exciting applications, streetlights play a vital role in our environment and also play a critical role in providing light for safety during night-time travel. In this scenario, when the streetlights are in a working position over the whole night, which consumes much energy and reduce the lifetime of the electrical appliances such as a light-emitting diode (LED) lamp, incandescent light bulb, gas discharge lamp, and high-intensity discharge lamps. Especially in cities’ streetlights, it is a severe power consuming factor and also the most significant energy expenses for a city. In this regard, an automation system is required to control the lights according to needs.

The traditional light system has been limited to two options: ON and OFF only, which are not efficient because these kinds of operations meant power loss due to continuing to work on maximum voltage. With the negligence of the operator or by some other technical problems, streetlights are continuously kept ‘ON’, even when there is no light required on the streets and this leads to the wastage of electricity. Hence, the wastage of power from street lights is one of the noticeable power losses, but with the use of automation, it leads to many new methods of energy and money saving. In this regard, controlling the lighting system using a light dependent resistor (LDR) [10], infrared radiation (IR) obstacle avoidance sensor [11], and Arduino [12,13] together are proposed in the past [14,15,16,17,18,19,20,21,22,23,24,25,26,27,28,29,30,31]. In previous literature, the street light systems are based on LDR [17,18,19,20,21,22,23,24,25,26,27,28,29], and most of them are passive infrared receiver-based systems that are controlled with timers and analogue circuits. Sun tracking sensors [32,33] are also used to power OFF the streetlights by the detection of the sunlight luminance. Furthermore, streetlight control with the use of solar energy [34,35], and ZigBee based system to control streetlights [36] have also been implemented. Distinguished from turning the electricity ON/OFF, another approach is introduced to DIM (half of the maximum brightness) the light [27,31] during the hours where traffic is sparse, which might be useful for reducing the power consumption, but with the electric bulbs under a continuous usage condition.

Apart from traditional home automation, the term “internet of things” (IoT) [23,37,38,39,40,41,42,43,44,45,46,47] is also important for connecting electrical appliances with internet that made it feasible to remotely control items from anywhere and anytime. After the introduction of IoT, the wireless systems provided a great help for automation systems by using cloud networks and Wi-Fi etc. Similarly, many wireless systems are made by using Bluetooth and smartphones connections [48] that can only be used by a particular person, because the mobile phone is not assumed to be always at home. In addition, the recent version of Bluetooth appears to be under a good agreement for low-power home automation devices [49]. Meanwhile, a new automation system was introduced, in which both Bluetooth Low Energy (BLE) devices are used instead of smartphones, with a limited number of specifications, but with a good and secure transfer rate [50]. Most precisely, the choices of these systems are hard to implement in a real case with real roads and real streetlights, due to its very low range of wireless connectivity. To the best of our knowledge, a need still exists for the design of a sunlight-based system that supports the DIM light concept, connecting the power ON/OFF with the objects’ detection, monitoring objects passing through the road, and controlling the entrance door.

In this paper, we propose and experimentally demonstrate a design to construct an automation system based on night time detection of objects. In the proposed automation system, the streetlights will be automatically turned OFF during day-time, otherwise the lights will remain DIM at night-time and turn ON (maximum brightness) at the object’s detection. This work is accomplished with the proper arrangements of the microcontroller Arduino Uno, the IR obstacle avoidance sensor, LDR, and resistors. It is noticed that the DIM state in the proposed design also means the continuous working of electric appliances over the whole night. To overcome this issue, the previously designed system is further extended to construct a system based only on the detection of objects. In this regard, the streetlights will turn ON automatically based on the detection of objects, otherwise the streetlights will remain OFF. Meanwhile, an automatic door system is also introduced in this object-dependent design that will operate with a motor and an IR obstacle avoidance sensor. The motor will automatically open the door when an IR obstacle avoidance sensor detects objects in front of the door, and shut it when no objects are detected. In addition, a counter is set to count the number of objects passing through the road, which will be displayed on the serial monitor of Arduino IDE [12,13]. Thus, the proposed systems (both night and objects’ detection; object-dependent) are designed and demonstrated using a lab-scale prototype to show that the proposed designs can be easily implemented in large-scale in near future.

The rest of this paper is structured as follows. In Section 2, a concept of the automation system based on sunlight is presented with a detailed description of the electronic components used in the proposed automation system, based on night time detection of objects, and an automation system based only on the detection of objects. In addition, the experimental results for a lab-scale prototype are provided, and Section 3 concludes the paper.

## 2. Materials and Methods

**Figure 1:**
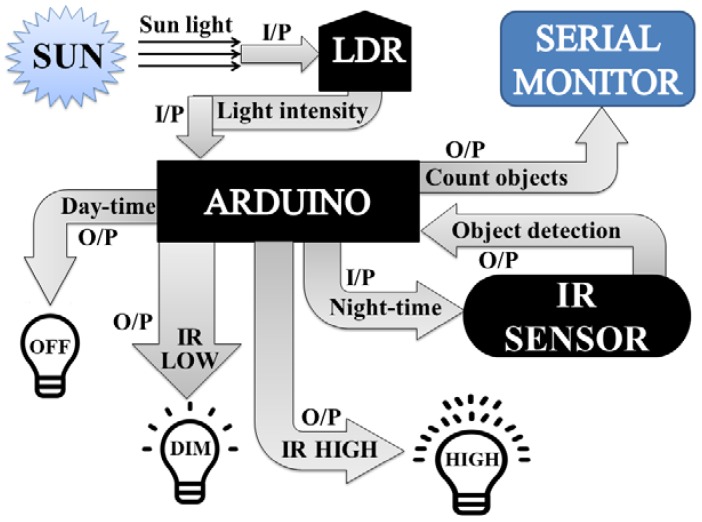
The architecture design of the automatic street light control system.

For the simplicity of discussion, Figure 1 illustrates the overall working mechanism and the features of the proposed lighting concept. I/P and O/P represents the input and output, respectively. LDR first senses the intensity value of sunlight and sends it to Arduino. After receiving the data, Arduino converts it into different discrete values from 0–1023, and judges whether the received value is above the threshold level (a limit value that is set independently by the user from the range of discrete values: 0–1023); it will then be considered as day-time, and the LEDs will remain OFF; if the received value is below the threshold level, Arduino will consider it as night-time. During night-time, if the value of IR obstacle avoidance sensor is LOW and detects no object, then DIM LEDs will glow, or if the IR obstacle avoidance value is HIGH and identifies any object, then HIGH LEDs will shine. Arduino also counts the total number of objects that cross the street in the night, with the help of IR obstacle avoidance sensor, and demonstrates this to the serial monitor.

### 2.1. Electronic Components

Multiple electronic components are used for building electronic circuits. Our proposed circuit designs contain these components that are described in Table 1.

#### 2.1.1. IR Obstacle Avoidance Sensor

An obstacle avoidance sensor is a heat sensitive sensor, and it is used for the detection of motion. It consists of an infrared-transmitter, an infrared-receiver and a potentiometer for adjusting the distance [54]. Whenever an object passes in front of a sensor, the emitted rays hit the surface of an object and reflect to the receiver of the sensor, and so it will consider this as a motion [11].

#### 2.1.2. LDR

The LDR [10] resistance is dependent on the amount of light impinging on it, and that resistance offered by the sensor decreases with an increase in light strength, and increases with a decrease in light intensity. LDR is used for the detection of day-time and night-time, because when sunlight falls on it, it will consider it as day-time, and when no sunlight falls on it, it will be regarded as night [55]. These are very beneficial, especially in light/dark sensor circuits, which help in automatically switching the street lights (ON/OFF).

#### 2.1.3. Arduino Uno

The Arduino Uno [12,13] (licensed under a Creative Commons Attribution Share-Alike 2.5) is a microcontroller board that is based on the ATmega328 series controllers, and has an integrated development environment (IDE) for writing, compiling, and uploading codes to the microcontroller. It has 14 digital input and output pins (of which six are pulse width modulation (PWM)) and six analog inputs for communication with the electronic components such as sensors, switches, motors, and so on. It also has 16 MHz ceramic resonators, a USB connection jack, an external power supply jack, an ICSP (in-circuit serial programmer) header, a reset button, GND pins (for grounding), and 5 V pin (for supplying 5 volts). Its operating voltage is 5 V, with an input voltage 7 to 12 V (limit up to 20 V).

#### 2.1.4. L298 Motor Module

An L298N motor module [53] is a heavy-duty dual H-bridge controller, which is used to control the direction and speed of single or two Direct Current (DC) motors simultaneously of up to 2 A each, having a voltage between 5 and 35 V DC—or one stepper motor. It has four output pins for connecting the DC motors, four input pins, two Enable jumpers (one of these corresponding jumpers can be removed and connected to the PWM output for DC motor speed control) [53]. It has an onboard 5 V regulator, so that if the supply voltage is up to 12 V, 5 V power will be given from the board.

### 2.2. Automation System Based on Night-Time Object Detection

**Figure 2:**
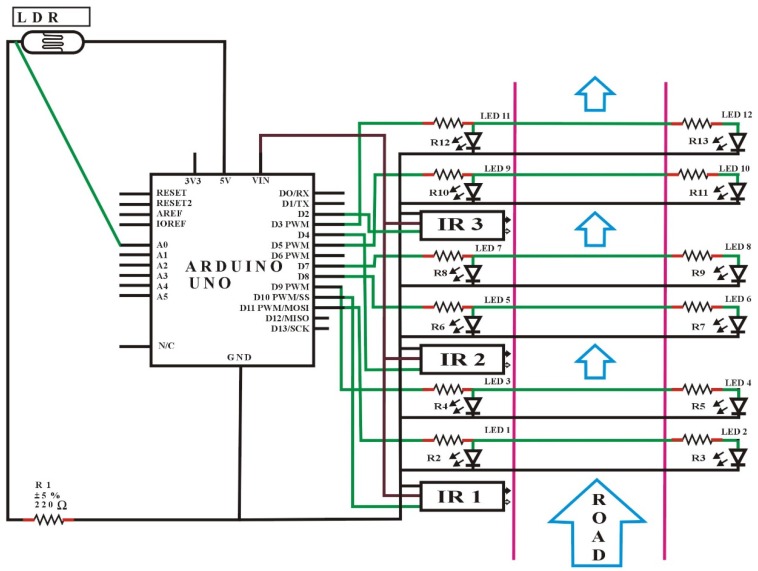
Circuit design of automation system based on night and objects’ detection.

Figure 2 shows the circuit design of an automatic street light control system based on object detection using Arduino Uno, with DIM light capability. In this scenario, the lights will turn to HIGH only with the detection of an object; otherwise the lights will remain OFF at day-time, and DIM at night-time.

In this task, an LDR sensor, 12 LEDs, 13 resistors, three IR obstacle avoidance sensors, and a single Arduino Uno were used. One leg of the LDR sensor was connected to Arduino analog pin number A0, and another leg to a 5 V pin, and the same with a resistor to the GND port of the Arduino. Besides, the threshold value for the LDR was adjusted to 10 from the discrete values (0–1023) for understanding whether it is day or night. After that, all the positive terminals of the LED set were connected with resistors to pin numbers D11, D9, D8, D7, D5, and D3 as the outputs of the Arduino signals. Here, one set of LEDs consists of two individual LEDs. Furthermore, the GNDs of all the LEDs were connected to the GND port, as shown in the circuit diagram (Figure 2).

The OUT terminals (represented by green lines of IR1, IR2, IR3) of the IR obstacle avoidance sensors were connected to the Arduino port from pin number D10, D4, and D2, respectively, which is the input signal to the Arduino board. Similarly, the GND of all the IR obstacle avoidance sensors were connected to the GND port, and all VCC (input voltage) of the IR obstacle avoidance sensors were connected to the Arduino 5 V pin. Initially, the IR obstacle avoidance sensors were set to LOW (by default) at the start, if there was no motion. Meanwhile, the detailed software code for this case is given in the Figure S1 of supplementary materials.

#### Results and Discussion

**Figure 3:**
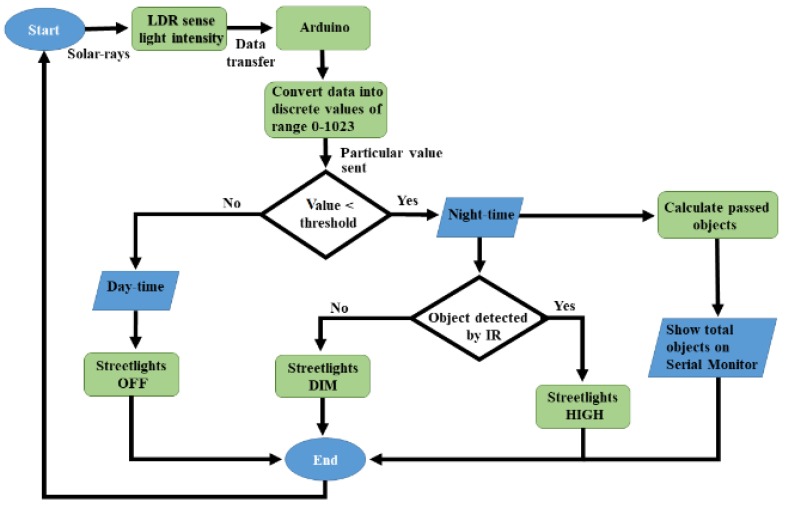
The flow diagram of the automation system based on night-time object detection.

In the beginning, the LDR sensor will sense the light intensity in the atmosphere at that time, and it will consequently transfer the data to Arduino, as can be seen in Figure 3. After receiving the data, Arduino will convert it into different discrete values from 0 to 1023 (in which 0 represents maximum darkness, and 1023 represents maximum brightness), and then it will adjust the output voltage accordingly from 0 to 2.5 V/5 V (DIM/HIGH) depending upon the received value (0–1023) by comparing it with the threshold value. Whereas, the threshold value can be randomly chosen by the user and in this case, the threshold value is adjusted to 10. So, the output will be 2.5 V in the complete darkness (night time), if the received value is less than the threshold value. As a result, DIM LEDs will glow that is the half of maximum brightness, and when there is completely shine (daytime), the received value will be higher than the threshold value, and the output voltage would be 0 V, resulting the LEDs to be completely switched OFF.

Initially, the IR obstacle avoidance sensor will be LOW. So, when there is no object in front of the sensor, the IR transmitter does continuously transmit the IR light. Whenever a car or any other object blocks any of the IR obstacle avoidance sensors, then the emitted rays will reflect the IR receiver after hitting the object, then microcontroller will sense it as a motion. In simple words, when an object passed in front of the first IR obstacle avoidance sensor, the corresponding LEDs will be turned from DIM to HIGH (5 V) by the microcontroller. As the object moves forward and blocks the next IR obstacle avoidance sensor, the next three LEDs will be turned to HIGH from DIM, and the LEDs from the previous set switched to DIM from HIGH. The process continues this way for the entire IR obstacle avoidance sensors and LEDs.

**Figure 4:**
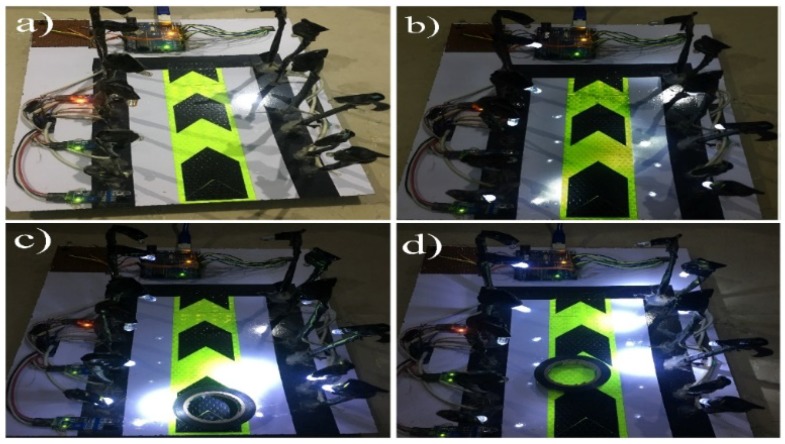
Result diagrams of the automatic streetlight control system that switches to DIM at night and HIGH at object detection. (**a**) In the day-time simulation, the LEDs are not glowing. (**b**) In the night-time representation, the DIM LEDs are glowing. (**c**) An object in front of first IR obstacle avoidance sensor; the first set of HIGH LEDs are glowing while the remainder are in DIM mode. (**d**) Motion in front of the second IR obstacle avoidance sensor; only the second set of LEDs are glowing HIGH, and all the others are in a DIM state.

Figure 4 shows the final demonstration of the proposed automatic streetlights system that turned to DIM at night-time and HIGH on vehicle movement using Arduino Uno. Figure 4a represents day-time, with no LEDs glowing after measuring the sensed intensity value of sunlight with the threshold value (10) by the LDR sensor. Figure 4b shows the night-time because the sensed intensity value of sunlight by LDR was below than the threshold value (10) and there was no motion detected by any of IR obstacle avoidance sensors, so as a result, the DIM LEDs were glowing. The beauty of the proposed model can be seen in Figure 4c,d, with the motive that only those LEDs that detect the object’s presence will glow brighter, and the remaining LEDs will keep maintaining their DIM state. As an example, in Figure 4c, the first set of LEDs are glowing HIGH, and remaining are in DIM mode because the sensed intensity value of sunlight by LDR is below then the threshold value, so it considered it to be night-time, and there was an object that was detected by the first IR obstacle avoidance sensor. Moreover, when the object moved to the second IR obstacle avoidance sensor, the second set of HIGH LEDs were glowing, and the first set again reverted to the DIM state (Figure 4d). These results demonstrate the efficiency of the proposed idea and give immediate validation for the proposed model. These kinds of application can be implemented in the headlights of objects, street lights, the parking lights of hotels, and in malls and homes.

### 2.3. An Automation System Based on Object Detection

**Figure 5:**
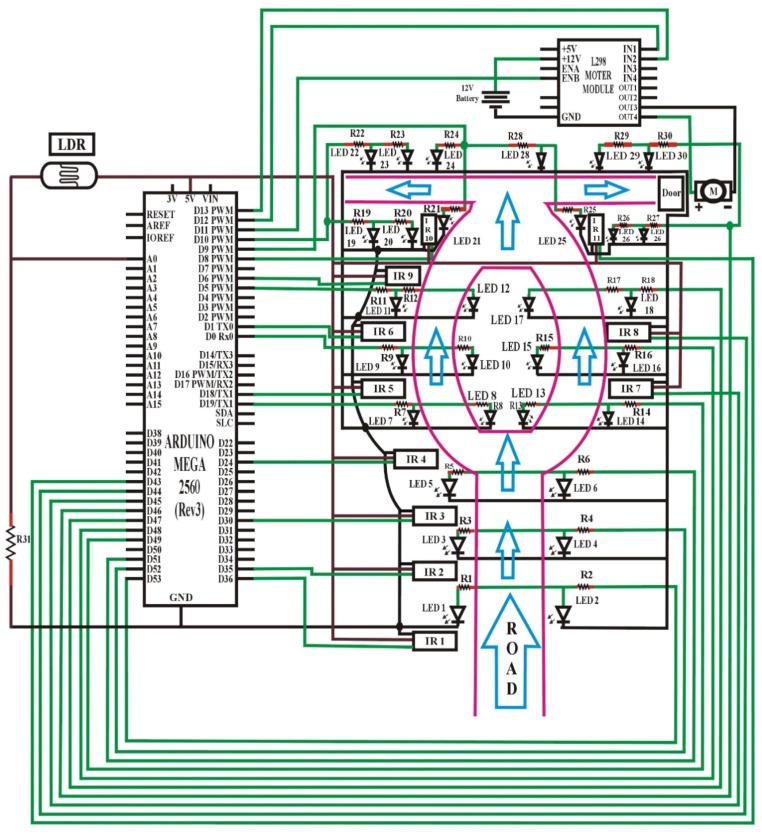
Circuit design of the automation system based on object detection and an automatic door system.

As per our motive, the main idea of this paper is to create such an innovation for our current street light system so that the power consumption can be saved. As presented in Figure 3, when there are no objects on the road at night-time, still, the DIM light continuously glows, and it wastes energy. Thus, the task is enhanced by controlling the streetlights based on object detection only at night-time. When the object is detected at night, the LEDs will switch ON automatically; otherwise the lights will remain OFF. The circuit design at lab-scale prototype can be seen in Figure 5. Most importantly, there is also an automatic door system in this design that will operate with a motor and an IR obstacle avoidance sensor. The motor will automatically open the door when an IR obstacle avoidance sensor detects any object in front of the door, and shut it when no objects are detected.

Figure 5 shows the circuit design of an automatic streetlight control system without DIM lights capability based on object detection using Arduino Mega [56]. In this design, an LDR sensor, 30 LEDs, 31 resistors, 11 IR obstacle avoidance sensors, a DC motor, an L298 motor module, and a single Arduino Mega were used. Hence, one leg of the LDR sensor was connected to the Arduino analog pin number A0, and another leg to a 5 V pin, and the same with a resistor to the GND port of Arduino. The threshold value was adjusted to 10 from discrete values (0–1023) for understanding whether it is day or night. All of the positive terminals of the LEDs set were connected, along with resistors to the pin number D53–D51, D19, D0, D5, D49–D47, D9, D10, and D46. In this regard, one set of LEDs consisted of two individual LEDs or more. Also, the GNDs of all the LEDs were connected to the GND port.

The OUT terminals (represented by green lines of IR1, IR2 and so on) of the IR obstacle avoidance sensors were connected to the Arduino port from pin number D36, D35, D30, D24, D18, D1, D45, D44, D6, D8, and D43 respectively, which was the input signal to the Arduino board. The GNDs of all of the IR obstacle avoidance sensors were connected to the GND port, and all the VCC (voltage at the common collector) of the IR obstacle avoidance sensors were directed in Arduino 5 V pin. Initially, the IR obstacle avoidance sensors were set to LOW (by default) at the start if there was no motion. Finally, the OUT-pin3 of the L298 motor module was connected with one side of the DC motor, and OUT-pin4 to another side. In this regard, the ENB (enable B) pin and IN (input) pins D1 and D2 were further attached to the pin numbers D11, D12, and D13 of Arduino, respectively. To supply the motor, a +12 V pin was connected to the positive terminal, and GND to the negative terminal of a 12 V battery. The detailed software code for this case is given in Figure S2 of the supplementary materials.

#### Results and Discussion

**Figure 6:**
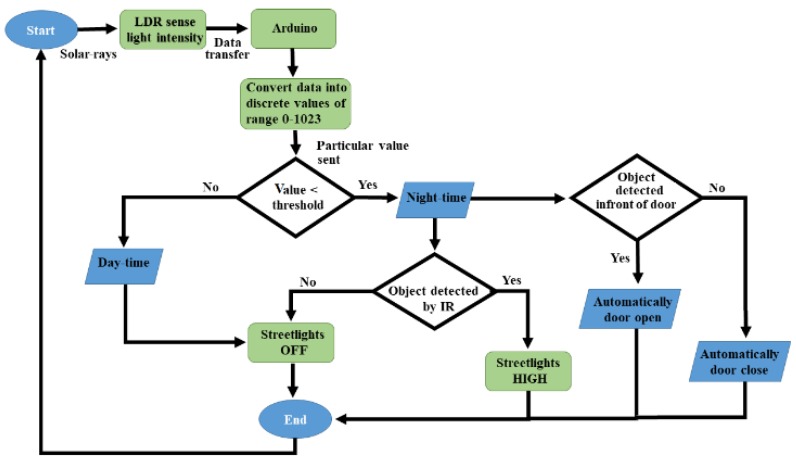
The flow diagram of the automation system based on object detection.

The functionality of the LDR sensor remained same as described in Figure 3, this can be further seen in Figure 6. In this scenario, the output will be 0 V in the complete darkness (night-time) if the received value is less than the threshold value and no LEDs will glow. Similarly, during day-time, the received value was higher than the threshold value, and the output was also 0 V, resulting in the LEDs being entirely switched OFF.

Initially, the IR obstacle avoidance sensor was LOW. Thus, when there was no object in front of the sensor, IR transmitter continuously transmitted IR rays. Whenever a car or any other object blocks any of the IR obstacle avoidance sensors, then the emitted rays will reflect the IR receiver after hitting the object, then microcontroller will sense it as a motion. In simple words, when an object passes in front of the first IR obstacle avoidance sensor, the corresponding LEDs will be turned from OFF to HIGH (5 V) by the microcontroller. As the object moves forward and blocks the next IR obstacle avoidance sensor, the next three LEDs will be turned to HIGH from OFF, and the LEDs from the previous set are switched to OFF from HIGH. The process continues this way for the entire set of IR obstacle avoidance sensors and LEDs.

**Figure 7:**
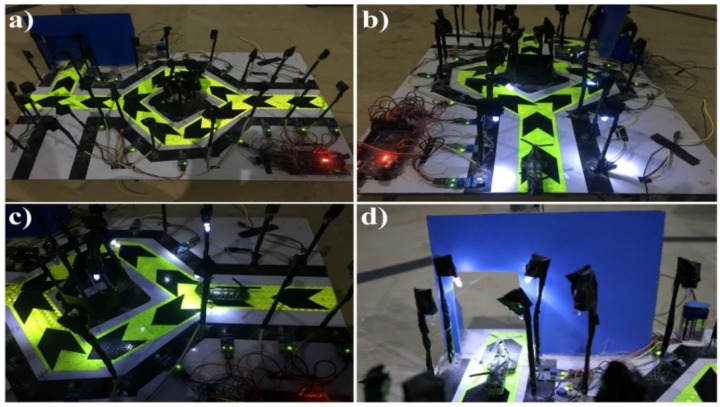
Result diagrams of enhanced work with an automatic door system and only an ON/OFF capability. (**a**) During a day/night-time simulation, the LEDs are not glowing. (**b**) Object in front of the first IR obstacle avoidance sensor; the HIGH LEDs are glowing. (**c**) Motion in front of the third IR obstacle avoidance sensor; the third set of LEDs are glowing. (**d**) An object detected is in front of the door, so it is automatically opened, and the relevant LEDs are glowing.

Figure 7 shows the result diagrams of the automatic streetlights system that turn to ON/OFF only, and having an automatic door system using the Arduino Mega Microcontroller. In this way, Figure 7a is represented with no LEDs glowing after the detection of day/night-time with the LDR sensor. On the other hand, in Figure 7b, the first set of HIGH LEDs glowed in the night-time because there was a motion that was detected by the first IR obstacle avoidance sensor. Similarly, when the object moved forward from the second IR obstacle avoidance sensor, then the third IR obstacle avoidance sensor detected its motion and the third set of HIGH LEDs glowed, with the remaining LEDs being turned OFF completely (Figure 7c). Meanwhile, when an object was detected by the IR obstacle avoidance sensor in front of the door, it automatically opened, and the corresponding lights also turned ON, as seen in Figure 7d. Table 2 summarizes the comparison of old systems and the proposed systems as a quick review.

We used the Arduino-based automation systems without any wireless connectivity, in which the streetlights could be automatically controlled based on solar rays, to avoid any limitations of the specifications, wireless range, etc. In some cases, we required wireless connectivity to make the system more scalable for easy integration of new devices, and online access to the information managed by the Arduino boards to develop a user-friendly interface. In this regard, the Ethernet network can be used to access more devices from internet, and an Ethernet shield would provide easy-to-use access to these data. Meanwhile, to store and secure the acquired information of devices for further study and analysis, different kind of database could be used in our proposed designs; in fact, Arduino is easy to integrate with a number of databases. Moreover, the reported systems are presented as lab-scale prototypes, as aforementioned; therefore, the managed power can be adjusted for real-scale facilities by driving an external voltage controller. Similarly, the proposed systems can be managed for real-scale facilities by applying other technologies such as BLE [57]. For the question of wireless connectivity, the sensors used in Figure 2 and Figure 6 can be replaced with different sensor types, or a combination of several types, for better results. For example, ultrasonic distance sensors [58] with the maximum range of 4 m range can be used; they are low cost and accurate enough for this kind of application. Alternatively, one can choose a long-range infrared-sensor [59] with a maximum range of 1.5 m, or a combination of laser diodes [60] and photo-resistors [10,55].

**Figure 8:**
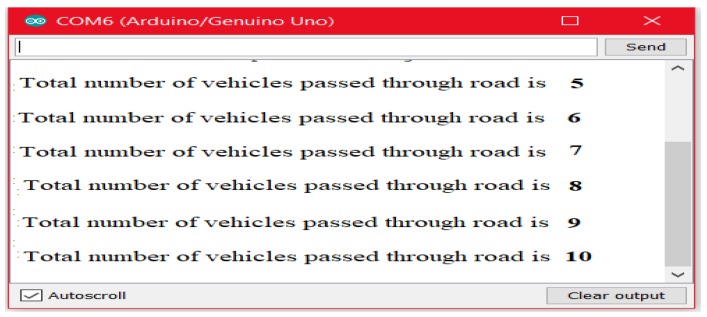
Serial monitor output according to traffic flow.

It is worth noting that the objects passing through the road often contain a large number of quantities that become more difficult to monitor at night-time. Thus, on the basis of monitoring objects to improve safety measurements, the serial monitor of Arduino IDE [12,13] is further adopted and considered as an interface to count and display the number of objects passing through the road at night-time. In this regard, when any object is detected by IR sensor (Figure 3), the Arduino will consider it as a vehicle movement and will demonstrate it on the serial monitor. Figure 8 illustrates the total number of objects on the serial monitor that passed through the road at night-time.

## 3. Conclusions

In this paper, a design scheme for controlling a streetlight system based on Arduino Uno microcontroller has been demonstrated, which can be programmed to react to events (based on night and object’s detection as described above) and to cause corresponding actions. The proposed scheme provided with two operational modes, in which the first automated system is used to control the streetlights based on night (lights turn to DIM state) and object detection (lights turn to HIGH state). The same system is further extended to design a second mode that turns the streetlights ON, based on only object’s detection. Meanwhile, it is presented that the proposed automated systems have capabilities to control the status of doors (closed/opened) and monitor objects. The hardware implementations of the proposed systems were carried out at a lab-scale prototype to verify the simplicity, flexibility, reliability, specificity and low cost of the system. As a lesson learned, we found that the proposed systems can be easily tested under real conditions at large-scale in near future, and it can be easily implemented in smart cities, home automation, agriculture field monitoring, timely automated lights, parking lights of hospitals, malls, airport, universities, and industries, etc.

## Figures and Tables

**Table 1:** Specification of electronic components used in to design the proposed system.

Components	Specifications
IR obstacle avoidance sensor [11]	Voltage: DC 3–5 V; Range 2–30 cm; Angle: 35°
LDR [10]	Voltage: DC 3–5 V; Diameter: 5 mm
Arduino Uno [12,13]	22 pins; Operating voltage: 6–20 V
LEDs [51]	Diameter: 5 mm; Operating voltage: 5 V
Resistors [52]	100 ohm; 220 ohms
L298 Motor Module [53]	Operating voltage: 5 V; Max power: 25 W

**Table 2:** Comparison between old systems and the proposed system.

Functionality	Old Systems	Proposed System
Based on Arduino	Yes	Yes
Operates with LDR and IR	Yes	Yes
DIM/FULL light capability with object detection	No	Yes
Displays the total number of objects passing through a road	No	Yes
Automatic door system	No	Yes

## Supplementary Materials

The following are available online at http://www.mdpi.com/1424-8220/18/10/3178/s1, Figure S1: Circuit design of automation system based on night and objects’ detection, Figure S2: Circuit design of automation system based on objects’ detection and having automatic door system.
