# Supplementary material for: An Automation System for Controlling Streetlights and Monitoring Objects Using Arduino
Source: Sensors (Basel). 2018 Sep 20;18(10):3178. doi: 10.3390/s18103178 (PMC6210980; doi:10.3390/s18103178)
Supplement: Supplementary file 1 [file sensors-18-03178-s001.pdf]

An Automation System for Controlling Streetlights and Monitoring Objects Using Arduino

Zain Mumtaz[1], Saleem Ullah[1], Zeeshan Ilyas[1], Naila Aslam[1], Shahid Iqbal[2], Shuo Liu[2], Jehangir Arshad Meo[3], Athar Mahboob[4] and Hamza Ahmad Madni[2,4*]

[1] Department of Computer Science, Khwaja Fareed University of Engineering & Information Technology, Rahim Yar Khan, 64200, Pakistan; zainmumtaz007@gmail.com (Z. M.); saleem.ullah@kfueit.edu.pk (S.U.); zeeshanilyas002@gmail.com (Z.I.); nailaaslam163@gmail.com(N.A.)

[2] State Key Laboratory of Millimeter Waves, Department of Radio Engineering Southeast University, Nanjing 210096, China; shahid@seu.edu.cn (S.I.); liushuo.china@seu.edu.cn (S.L.)

[3] Department of Electrical Engineering, COMSATS University Islamabad, Islamabad 45550, Pakistan; jehangir@ciitsahiwal.edu.pk (J.A.M.)

[4] Department of Computer Engineering, Khwaja Fareed University of Engineering & Information Technology, Rahim Yar Khan, 64200, Pakistan; athar@kfueit.edu.pk (A.M.)

* Correspondence: 101101770@seu.edu.cn (H.A.M); Tel.: +86-1782-6500-615 / +92-313-4820-856 (H.A.M)

Supplementary Material

Automation System Based on Night and Objects' Detection

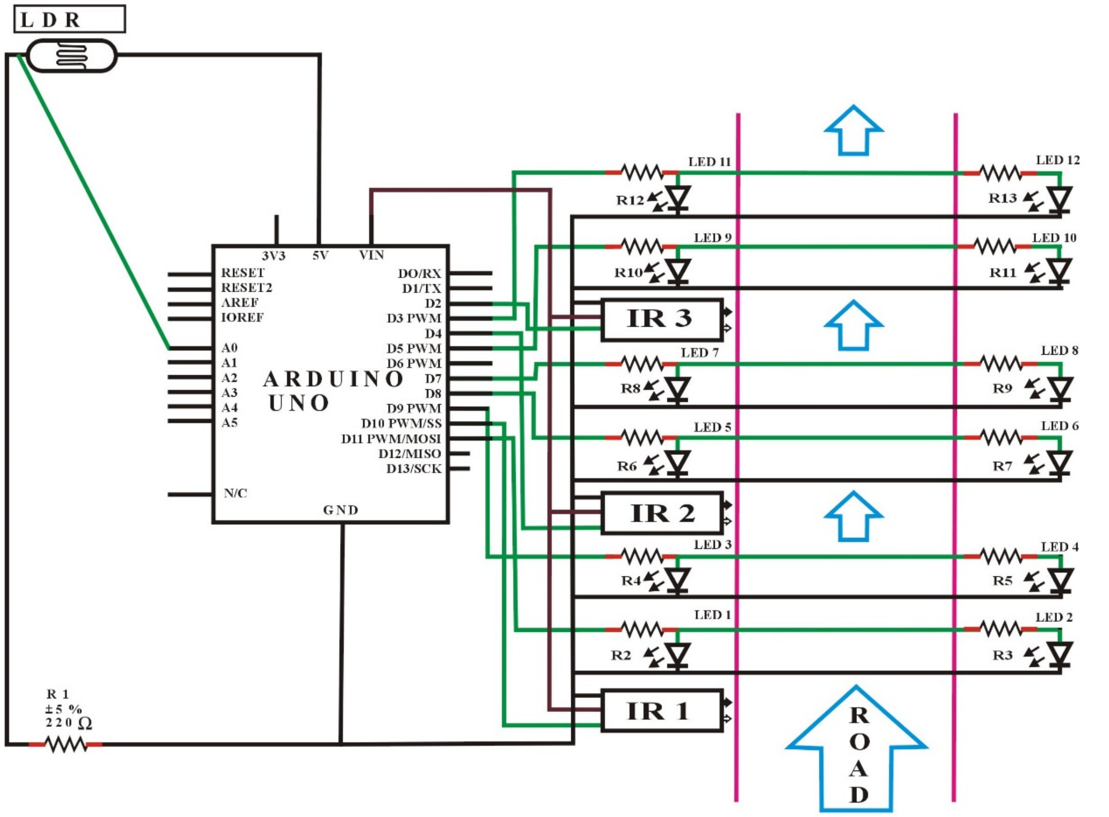

Figure S1. Circuit design of automation system based on night and objects' detection.

Figure S1 shows the circuit design of automatic street light control system based on vehicle detection using Arduino Uno having feature of DIM light capability. All the explanation of this system has been described in the Figure 2 of main manuscript and the code is given as:

```
int LED1 = 11;
int LED2 = 9;
int LED3 = 8;
int LED4 = 7;
int LED5 = 5;
int LED6 = 3;
int i1 = 10;
int i2 = 4;
int i3 = 2;
int proxy1=0;

int proxy2=0;
int proxy3=0;
int a=0;
int hasObstacle = HIGH;
int dim = 15;
int full = 255;
int LDR = A0;
int LDRReading = 0;
int threshold_val = 10;
void setup() {
  pinMode(LED1, OUTPUT);
  pinMode(LED2, OUTPUT);
  pinMode(LED3, OUTPUT);
  pinMode(LED4, OUTPUT);
  pinMode(LED5, OUTPUT);
  pinMode(LED6, OUTPUT);
  pinMode(i1, INPUT);
   pinMode(i2, INPUT);
   pinMode(i3, INPUT);
   Serial.begin(9600);
  }
```

```
void loop() {
  LDRReading = analogRead(LDR);
  Serial.println(LDRReading);
  proxy1=digitalRead(i1);
  proxy2=digitalRead(i2);
  proxy3=digitalRead(i3);
  if (LDRReading <threshold_val) {
            if (proxy1 == HIGH) {
              a=a+1;
      analogWrite(LED1,full);
      analogWrite(LED2,full);
   }
  else
  {

 analogWrite(LED1,dim);
    analogWrite(LED2,dim);
    delay(100);
  }
            if (proxy2 == HIGH) {
      analogWrite(LED3,full);
      analogWrite(LED4,full);
  }
  else
  {
    analogWrite(LED3,dim);
    analogWrite(LED4,dim);
    delay(100);
  }
if (proxy3 == HIGH) {
      analogWrite(LED5,full);
      analogWrite(LED6,full);
  }
  else
  {
    analogWrite(LED5,dim);
```

```
    analogWrite(LED6,dim);
    delay(100);
  }
        Serial.println("Vehicles Passed Through Road");
      Serial.print(a);
  }
  else
{
  analogWrite(LED1, 0);
  analogWrite(LED2, 0);
  analogWrite(LED3, 0);
  analogWrite(LED4, 0);
  analogWrite(LED5, 0);
  analogWrite(LED6, 0);
  }
 }
```

Automation System Based on Objects' Detection

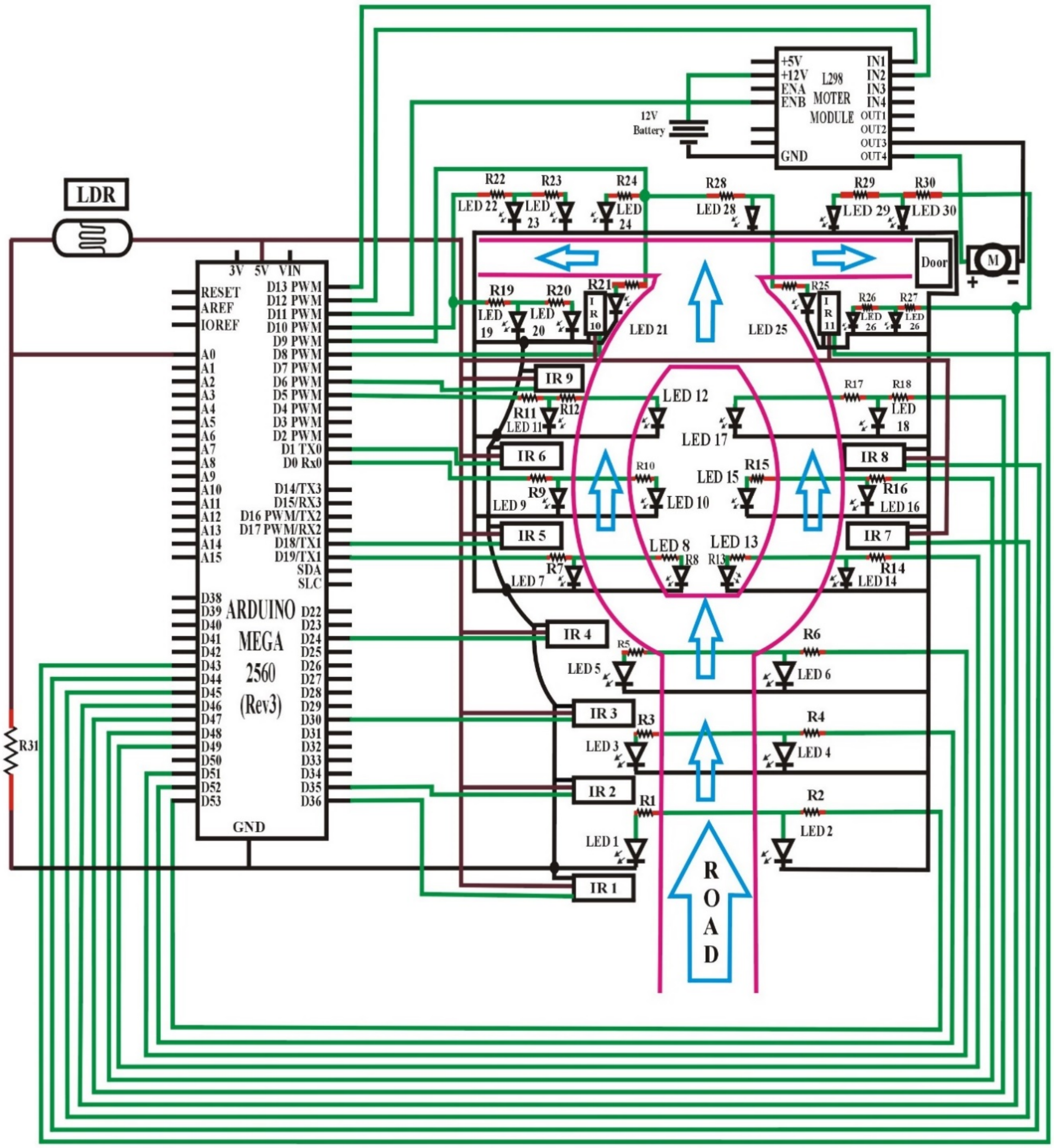

Figure S2. Circuit design of automation system based on objects' detection and having automatic door system.

Figure S2 shows circuit design of automatic streetlight control system without DIM LEDs capability based on Vehicle Detection Using Arduino Mega with an automatic door system. All the explanation of this system has been described in the Figure 5 of main manuscript and the code is given as:

```
int ir1=36; // IR PINS
int ir2=35;
int ir3=30;
int ir4=24;
int ir5=18;
int ir6=1;
int ir7=45;
int ir8=44;
int ir9=6;
int ir10=8;
int ir11=43;
int led1=53;  // LEDs PINS
int led2=52;
int led3=51;
int led4=19;
int led5=0;
int led6=5;
int led7=49;
int led8=48;
int led9=47;
int led10=9;
int led11=10;
int led12=46;

int proxy1=0;       //LEDs Function
int proxy2=0;
int proxy3=0;
int proxy4=0;
int proxy5=0;
int proxy6=0;
int proxy7=0;
int proxy8=0;
int proxy9=0;
```

```
int proxy10=0;
int proxy11=0;
int a=0;
int hasObstacle = HIGH;
int dim = 15;
int full = 255;
int LDR = A0;
int LDRReading = 0;
int threshold_val = 10;
void setup()
{
  pinMode(ir1,INPUT);
  pinMode(ir2,INPUT);
  pinMode(ir3,INPUT);
  pinMode(ir4,INPUT);
  pinMode(ir5,INPUT);
  pinMode(ir6,INPUT);
  pinMode(ir7,INPUT);
  pinMode(ir8,INPUT);
  pinMode(ir9,INPUT);
  pinMode(ir10,INPUT);
  pinMode(ir11,INPUT);
  pinMode(led1,OUTPUT);
  pinMode(led2,OUTPUT);
  pinMode(led3,OUTPUT);
  pinMode(led4,OUTPUT);
  pinMode(led6,OUTPUT);
  pinMode(led7,OUTPUT);
  pinMode(led8,OUTPUT);
  pinMode(led9,OUTPUT);
  pinMode(led10,OUTPUT);
  pinMode(led11,OUTPUT);
  pinMode(11,OUTPUT); // motersss
  pinMode(12,OUTPUT);
  pinMode(13,OUTPUT);
  Serial.begin(9600);
```

```
}
void loop(){
  LDRReading = analogRead(LDR);
  Serial.println(LDRReading);
  proxy1=digitalRead(ir1);
  proxy2=digitalRead(ir2);
  proxy3=digitalRead(ir3);
  proxy4=digitalRead(ir4);
  proxy5=digitalRead(ir5);
  proxy6=digitalRead(ir6);
  proxy7=digitalRead(ir7);
  proxy8=digitalRead(ir8);
  proxy9=digitalRead(ir9);
  proxy10=digitalRead(ir10);
  proxy11=digitalRead(ir11);
if (LDRReading <threshold_val) {
if(proxy1==HIGH)
              {
                digitalWrite(led1,HIGH);
                digitalWrite(led2,HIGH);
                                a=a+1;
                delay(1000);
              }
            else
            {
              digitalWrite(led1,LOW);
              digitalWrite(led2,LOW);
            }
if(proxy2==HIGH)
              {
                digitalWrite(led2,HIGH);
                digitalWrite(led3,HIGH);
                 delay(1000);
              }
              else
              {
```

```
              digitalWrite(led2,LOW);
               digitalWrite(led3,LOW);
             }
if(proxy3==HIGH)
               {

                 digitalWrite(led3,HIGH);
                 digitalWrite(led4,HIGH);
                 digitalWrite(led7,HIGH);
                  delay(1000);
               }
               else
               {
                 digitalWrite(led3,LOW);
                 digitalWrite(led4,LOW);
                 digitalWrite(led7,LOW);
               }
if(proxy4==HIGH)
               {
                 digitalWrite(led4,HIGH);
                 digitalWrite(led5,HIGH);
                  delay(1000);
               }
               else
               {
                 digitalWrite(led4,LOW);
                 digitalWrite(led5,LOW);
               }
 if(proxy5==HIGH)
               {
                 digitalWrite(led5,HIGH);
                 digitalWrite(led6,HIGH);
                  delay(1000);
               }
               else
               {
```

```
                digitalWrite(led5,LOW);
                digitalWrite(led6,LOW);
              }
if(proxy6==HIGH)
              {
                digitalWrite(led6,HIGH);
                 delay(1000);
              }
              else
              {
                digitalWrite(led6,LOW);
              }
if(proxy7==HIGH)
              {
                digitalWrite(led8,HIGH);
                digitalWrite(led9,HIGH);
                 delay(1000);
              }
              else
              {
                digitalWrite(led8,LOW);
                digitalWrite(led9,LOW);
              }
if(proxy8==HIGH)
              {
                digitalWrite(led9,HIGH);
                 delay(1000);
              }
              else
              {
                digitalWrite(led9,LOW);
               }
if(proxy9==HIGH)
              {
                digitalWrite(led10,HIGH);
                 delay(1000);
```

```
        }
        else
        {
          digitalWrite(led10,LOW);
         }
if(proxy10==HIGH)
        {
          digitalWrite(led11,HIGH);
           delay(1000);
        }
        else
        {
          digitalWrite(led11,LOW);
         }
if(proxy11==HIGH)
        {
          digitalWrite(led12,HIGH);
           delay(1000);
        }
        else
        {
          digitalWrite(led12,LOW);
         }
        Serial.println("Vehicles Passed Through Road");
        .print(a);
                }
else{
  analogWrite(LED1, 0);
  analogWrite(LED2, 0);
  analogWrite(LED3, 0);
  analogWrite(LED4, 0);
  analogWrite(LED5, 0);
  analogWrite(LED6, 0);
  analogWrite(LED7, 0);
  analogWrite(LED8, 0);
  analogWrite(LED9, 0);
```

```
  analogWrite(LED10, 0);
  analogWrite(LED11, 0);
  analogWrite(LED12, 0);
  }
}
```
